# The Effects of Spinal Manipulation on Motor Unit Behavior

**DOI:** 10.3390/brainsci11010105

**Published:** 2021-01-14

**Authors:** Lucien Robinault, Aleš Holobar, Sylvain Crémoux, Usman Rashid, Imran Khan Niazi, Kelly Holt, Jimmy Lauber, Heidi Haavik

**Affiliations:** 1 Laboratoire d’Automatique, de Mécanique et d’Informatique Industrielles et Humaines (LAMIH UMR CNRS 8201), Université Polytechnique Hauts-de-France, F-59313 Valenciennes, France; lucien.robinault@uphf.fr (L.R.); jimmy.lauber@uphf.fr (J.L.); 2 Faculty of Electrical Engineering and Computer Science, University of Maribor, SI-2000 Maribor, Slovenia; ales.holobar@um.si; 3 Centre de Recherche Cerveau et Cognition, Université de Toulouse, UPS, 31052 Toulouse, France; sylvain.cremoux@cnrs.fr; 4 The Brain and Cognition Research Center (CerCo), CNRS UMR 5549, 31052 Toulouse, France; 5 Health and Rehabilitation Research Institute, AUT University, Auckland 0627, New Zealand; usman.rashid@aut.ac.nz; 6 Centre for Chiropractic Research, New Zealand College of Chiropractic, Auckland 1060, New Zealand; kelly.holt@nzchiro.co.nz (K.H.); heidi.haavik@nzchiro.co.nz (H.H.); 7 Department of Health Science and Technology, Aalborg University, 9220 Aalborg, Denmark

**Keywords:** high-density surface electromyography, chiropractic, electromyography decomposition, motor unit

## Abstract

Over recent years, a growing body of research has highlighted the neural plastic effects of spinal manipulation on the central nervous system. Recently, it has been shown that spinal manipulation improved outcomes, such as maximum voluntary force and limb joint position sense, reflecting improved sensorimotor integration and processing. This study aimed to further evaluate how spinal manipulation can alter neuromuscular activity. High density electromyography (HD sEMG) signals from the tibialis anterior were recorded and decomposed in order to study motor unit changes in 14 subjects following spinal manipulation or a passive movement control session in a crossover study design. Participants were asked to produce ankle dorsiflexion at two force levels, 5% and 10% of maximum voluntary contraction (MVC), following two different patterns of force production (“ramp” and “ramp and maintain”). A significant decrease in the conduction velocity (*p* = 0.01) was observed during the “ramp and maintain” condition at 5% MVC after spinal manipulation. A decrease in conduction velocity suggests that spinal manipulation alters motor unit recruitment patterns with an increased recruitment of lower threshold, lower twitch torque motor units.

## 1. Introduction

Over recent years, a growing body of research has investigated the neurophysiological effects of spinal manipulation [1,2].

These studies revealed that spinal manipulation improves outcomes, such as maximum voluntary contraction and joint position sense [3,4,5,6,7], and that these changes are associated with central plastic adaptations in sensorimotor integration at the cortical level [3,7,8,9,10,11].

Two previous studies have reported that participants receiving a single session of spinal manipulation [5], or 12 weeks of chiropractic care [6], showed an improvement in elbow or ankle joint position sense, respectively, in comparison to control participants. This suggests that spinal manipulation has an impact on the integration and processing of somatosensory information from the limbs. Other studies have used electroencephalography (EEG) to measure sensory integration adaptation at the cortical level after spinal manipulation and have reported that early cortical somatosensory evoked potentials (SEPs), such as N20 and N30, significantly decreased after cervical spinal manipulation in a relatively healthy population [12,13,14]. These early cortical SEP peaks are thought to represent the arrival of the afferent signals at the primary somatosensory cortex and early sensorimotor integration, respectively [15,16]. Interestingly, in a chronic stroke population, who are known to have a reduced amplitude of early SEP peaks [17], a recent study showed an increase in N30 SEP peak amplitude following chiropractic care [18]. These observed changes in SEP peak amplitude after chiropractic care indicate that spinal manipulation can alter cortical somatosensory processing and results in neural plastic changes in the central nervous system (CNS) [11].

Motor control changes following spinal manipulation have also previously been documented [4]. One study showed that spinal manipulation altered cortical motor control of two upper limb muscles in a muscle-specific manner [14]. This study used a paired-pulse transcranial magnetic stimulation (TMS) protocol to explore specific, central corticomotor facilitatory, and inhibitory neural pathways to the two upper limb target muscles. After spinal manipulation, there was a decrease in short interval intracortical inhibition, an increase in intracortical facilitation, and a shortening of the cortical silent period in the flexor muscle, whereas the extensor muscle showed opposite changes, with a decrease in intracortical facilitation and a lengthening of the cortical silent period [14]. Another TMS study measuring stimulus response curves found that spinal manipulation led to changes in corticospinal excitability, as measured by a significantly larger motor evoked potential for both upper and lower limb muscles, and larger amplitudes of movement-related cortical potential (MRCP) components with no change in spinal F-wave amplitude or persistence [10], suggesting these changes occurred at the supra-spinal level [16].

Overall, these studies suggest that spinal manipulation alters central corticomotor processing. Interestingly, these central motor adaptations also influence motor behavior, as several studies have shown significant increases in maximum voluntary plantarflexion force production after a single session of spinal manipulation [3,7,19]. The first of these studies showed that the force increase was associated with a large increase in the cortical-based V-wave and a small, but significant, decrease in the spinal H-reflex threshold, respectively, reflecting supraspinal and spinal cord adaptative changes in α-motor neuron excitability [19]. Two follow-up studies in elite athletes [3] and chronic stroke patients [3] also showed increased ankle plantarflexion force production with a large V-wave increase following spinal manipulation, but no changes in the H-reflex. Overall, these results suggest that spinal manipulation alters motor preparation and commands from the supraspinal regions to more efficiently control force production. Combined, these findings suggest that spinal manipulation has an impact on central cortical processing that improves the accuracy with which the brain is aware of limb position and alters the way the brain controls the upper and lower limbs, leading to more efficient motor control [3,4,5,7]. Yet, how these central neural adaptations translate at the neuromuscular level remains to be studied.

Neuromuscular changes can be studied via the assessment of motor unit (MU) activity. The gold standard to study MU properties is the decomposition of intramuscular electromyographic (EMG) signals [20,21], but this technique is invasive and limits the area of the muscle that can be studied [22]. To overcome these drawbacks, high-density surface EMG (HD sEMG) has been developed, together with advanced signal processing techniques [23,24,25,26,27,28,29]. Numerous variables can be estimated after decomposition of the HD sEMG signals into MU action potentials (MUAP), such as MU firing rate, instantaneous MU firing rate gradient, and changes in the propagation velocity of the MUAP across the muscle fibers [30,31,32]. The MU firing rate refers to the instantaneous rate of coding of the MU [33]. The maximum firing rate value is known as the peak firing rate, while the instantaneous MU firing rate gradient refers to the derivative of the firing rate. The conduction velocity refers to the speed at which the neural impulse propagates from the motor unit through the muscle fibers [16].

The previous studies that have shown changes in ankle plantarflexion force, V-waves, and the H-reflex following spinal manipulation [3,7,19] have been conducted using a small number of surface EMG electrodes. This enables few conclusions to be made about changes in MU behavior associated with spinal manipulation [3,7,19]. To better understand how spinal manipulation influences MU behavior and neuromuscular control, HD sEMG can be used in place of standard surface electrodes [23,34]. The changes in motor control following spinal manipulation that have been observed in previous studies could be due to a shift towards activation of lower threshold MU’s [4]. If this is the case, and because conduction velocity of a MU is highly correlated to its twitch torque production, one would expect to see lower conduction velocity and higher peak firing rate values and an increase in instantaneous MU firing rate gradient after spinal manipulation [35]. Thus, to further explore the changes in motor unit behavior that occur after spinal manipulation, this study aimed to investigate neuromuscular adaptation of the tibialis anterior (TA) muscle before and after a single session of spinal manipulation in a subclinical pain population.

## 2. Materials and Methods

### 2.1. Experimental Design and Setting

**Figure 1:**
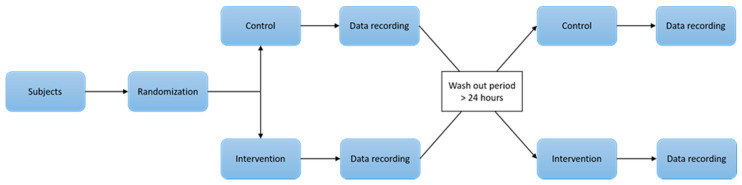
Illustration of the study design.

The experiment was conducted as a randomized, crossover study, where each subject participated in both control and intervention groups (see Figure 1).

The experiment took place at Aalborg University Hospital, Aalborg, Denmark. Before the experiment, the subjects were allocated randomly to either receive the chiropractic intervention or sham intervention first. Participants were then assessed before and after receiving the appropriate intervention. After a wash out period of at least 24 h, the participants were retested before and after receiving the alternate intervention. The study was conducted according to the Declaration of Helsinki. The North Denmark Region Committee on Health Research Ethics (N-20140027) approved the study. All participants gave written informed consent before participating in the study.

### 2.2. Participants

Fourteen participants (10 males and 4 females; age: 28.6 ± 2.8 years) were recruited via convenience sampling for this study. Participants were included if they presented with a history of mild back pain or neck pain for which they had not previously sought treatment and had evidence of mild spinal dysfunction based on a chiropractic assessment. Participants fulfilling these criteria can be referred to as having subclinical pain [8]. Subclinical pain (SCP) refers to recurring spinal dysfunction, such as mild pain, ache, and/or stiffness, with or without a history of known spinal trauma. Individuals with SCP do not have constant symptoms and have not yet sought treatment of their spinal problems. There is an increasing interest in SCP in the literature [5,8,36,37,38,39,40,41,42,43] because individuals that fall into this category provide an opportunity to explore neurophysiologic dysfunction without the confounding effect of current pain, nor the confounding effects of previous drug use or surgery. Changes in pain is known to alter sensory processing and motor control [44], as would various drugs and surgery. Studying this group may, therefore, help to gain a better understanding of the features of those developing more serious chronic pain states and may also help characterizing which subgroups of those developing pain problems that may respond to different sorts of treatment options.

On the day of the experiment, the participants were excluded from the study if they presented with a pain level greater than 2 on a 10-points pain scale [45]. Sample size calculations and predicted effect sizes were based on changes observed in a previous study that investigated similar neurophysiological changes before and after spinal manipulation (SM) in previous study Niazi et al. [19]. For an effect size of 0.5, 11 subjects were needed to be able to reject the null hypothesis that the population means of the experimental and control groups are equal with probability (power) 0.9. To allow for drop out during the trial and relative uncertainty relating to power outcomes, we aimed to enroll 15 subjects in the trial but were able to recruit 14.

### 2.3. Spinal Manipulation and Sham Intervention

The chiropractor assessed the entire spine and both sacroiliac joints for segmental dysfunction (also called vertebral subluxations by chiropractors). The clinical indicators that were used to assess joint function included assessing for tenderness to palpation of the relevant joints, manually palpating for restricted range of motion, assessing for palpable asymmetric muscle tension, and any abnormal or blocked joint play or end-feel of the joints. All of these biomechanical characteristics are used by chiropractors as clinical indicators of joint dysfunction [46].

For the spinal manipulation intervention, a New Zealand registered chiropractor checked participants for spinal dysfunction known as chiropractic or vertebral subluxations and adjusted them, where necessary. Vertebral subluxations are recognized as biomechanical lesions of the vertebral column by the World Health Organization (ICD-10-CM code M99.1) [47]. Due to the past two decades of basic science research, they are now viewed as a self-perpetuating, central segmental motor control problem that involves a joint, such as a vertebral motion segment, that is not moving appropriately, resulting in ongoing maladaptive neural plastic changes that interfere with the central nervous system’s (CNS’s) ability to self-regulate, self-organize, adapt, repair, and heal [48]. It is thought that such “maladaptation” of body posture may be beneficial and is used to avoid further pain from the region (pain adaptation concept) [49]; however, when maintained for a long period of time, they become maladaptive or harmful. The clinical indicators that were used to assess joint function, and to identify vertebral subluxations, included assessing for tenderness to palpation of the relevant joints, manually palpating for restricted range of motion, assessing for palpable asymmetric muscle tension, and any abnormal or blocked joint play or end-feel of the joints. These clinical indicators are routinely used by chiropractors when analyzing the spine and have previously been shown to be reliable for the identification of chiropractic subluxations when used as a multidimensional battery of tests [46,49].

During the chiropractic intervention, the chiropractor manipulated the areas of joint dysfunction using high velocity, low amplitude thrusts. All of the spinal manipulations carried out in this study were manual high-velocity, low-amplitude thrusts to the spine or pelvic joints [50]. This is a standard manipulation technique used by chiropractors, and is also referred to as spinal adjustments. The mechanical properties of this intervention have been investigated; and, although the actual force applied to the subject’s spine depends on the chiropractor, the patient, and the spinal location of the manipulation, the general shape of the force-time history of spinal manipulations is very consistent [51], and the duration of the thrust is always less than 200 milliseconds [52]. This high-velocity type of manipulation was specifically chosen because previous research has shown that reflex EMG activation is observed after high-velocity, low-amplitude manipulations but not lower-velocity mobilizations [53]. This manipulation technique has also been previously used in studies that have investigated the neurophysiological effects of spinal manipulation [4].

The control intervention was intended to act as a control treatment session, as well as to act as a physiological control for possible changes occurring due to the cutaneous, muscular, or vestibular input that would occur with the type of passive and active movements involved in preparing a subject/patient for a manipulation. During the control intervention, the subject’s head and/or spine and peripheral joints were moved passively and actively in ways similar to what was done by the chiropractor that provided the actual chiropractic intervention. Thus, the control intervention involved the subjects being moved into the manipulation setup positions similar to how the chiropractor would normally setup a subject prior to applying a manipulative thrust. Loading a joint, as is done prior to spinal manipulation, has been shown to alter paraspinal proprioceptive firing in anesthetized cats [54] and, therefore, was carefully avoided by ending the movement prior to end range of motion when passively moving the subjects. No spinal manipulation was performed during any control intervention.

For each subject, the chiropractor performing the real and control interventions was the same and had at least 10 years of clinical experience.

### 2.4. Procedure

The foot of the participant was placed in an ankle ergometer, at a neutral angle, with a strap to fix the foot in place at the metatarsal level. At the beginning and the end of the experimental protocol, the participants were asked to perform 3 dorsiflexion maximal voluntary contractions (MVCs) of 3 s, with 2 min rest in between each MVC.

**Figure 2:**
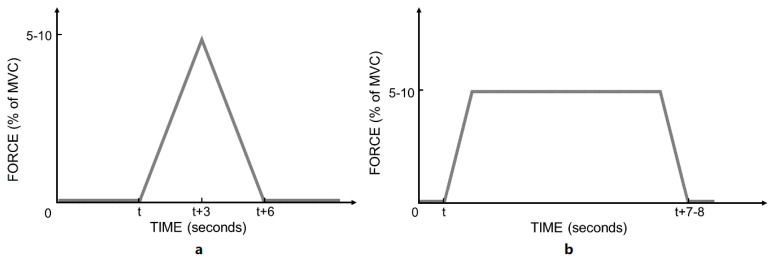
Force pattern for the subject to follow. (**a**) “Ramp” condition; (**b**) “ramp and maintain” condition.

The participants were then asked to produce dorsiflexion following a triangular “ramp” or trapezoidal “ramp and maintain” force production pattern, as described in Figure 2. The “ramp” condition consisted of a 3 s increase of force production to reach the target force intensity, then 3 s ramping down to rest. The “ramp and maintain” condition consisted of a ramping up phase of 1 s to the target force intensity, then maintaining this intensity for 6 s, followed by a ramping down phase of 1 s to rest. The force intensity was either 5% or 10% of the MVC recorded at the beginning of the experiment. The reasons to use low % MVC was to specifically investigate the low threshold single motor units because only the threshold of the H-reflex (i.e., reflecting low threshold motor units) was shown to change following a spinal manipulation intervention by Niazi et al. [19].

The purpose of the “ramp” condition was to find the firing threshold of the detected MUs and the “ramp & maintain” condition was used to “isolate” MU’s with different firing thresholds [55,56,57]. The force intensity of 5% and 10% was chosen as the HD sEMG decomposition techniques used are reliable at these force levels [58] and because previous research suggested, via indirect measures, that spinal manipulation is more likely to have an impact on lower threshold MUs [19].

Before and after the interventions, the subjects executed 3 contractions following the “ramp” condition pattern at the first intensity of contractions, and then at the second intensity of contractions. The first force intensity was randomly determined for each participant using computer-generated random allocation. The subjects then executed 3 contractions following the “ramp and maintain” condition pattern at each intensity of contractions. Contractions were separated by a 10 s rest period. Conditions were separated by a 5-min break.

### 2.5. Equipment

#### 2.5.1. Ankle Ergometer

Force was recorded from a force transducer [19] mounted on a custom-made pedal. The force data were also displayed on a screen in front of the subject and used in real time to provide the target pattern and force level that the subject had to develop. The force was sampled at 2000 Hz and acquired with ‘Mr. Kick’ software (SMI, Aalborg University, Aalborg, Denmark).

#### 2.5.2. HD EMG

**Figure 3:**
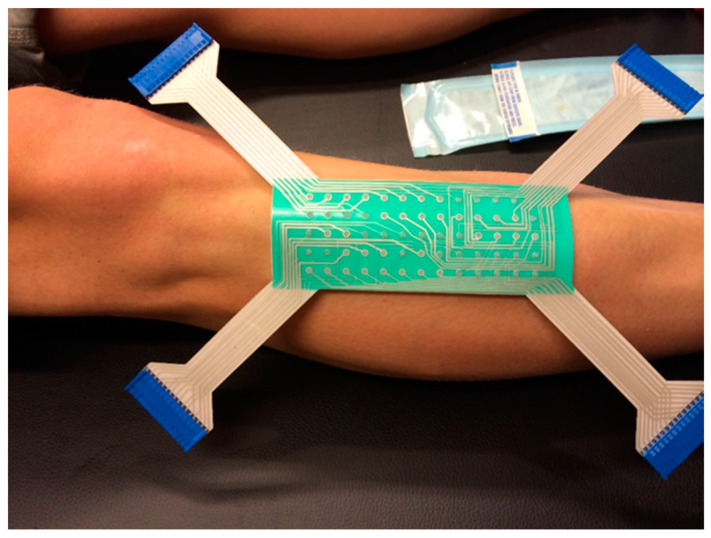
Placement of the ELSCH064R3 electrode grid; note the missing electrode on the top right corner.

**Figure 4:**
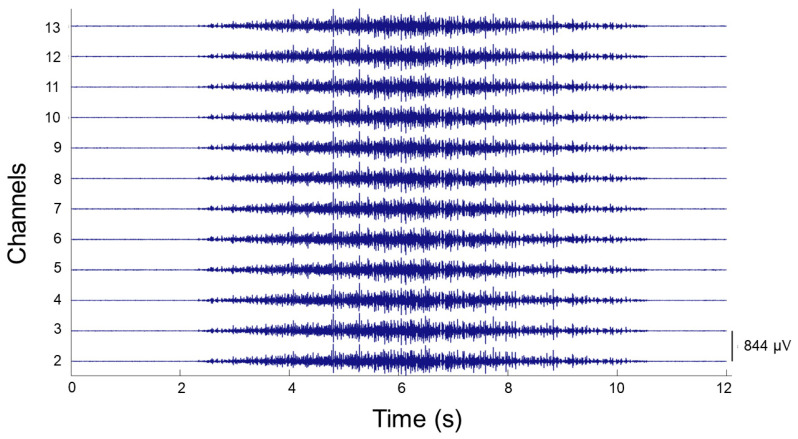
Representative electromyographic (EMG) signals from the first row of electrodes of the ELSCH064R3 (OT-Bioelectronica, Italy) grid, acquired on subject 2, before control intervention during a “ramp” condition task at 10% maximum voluntary contraction (MVC).

EMG signals from the tibialis anterior (TA) muscle were recorded with semi-disposable adhesive 64 sensor grid matrix electrodes (ELSCH064R3, OT Bioelettronica, Torino, Italy) coupled to an amplifier (EMG-USB2+, OT Bioelettronica). The grid was applied on the dominant leg of the subject after the skin had been abraded with a paste (Meditec-Every, Parma, Italy). The position of the *missing* electrode was at the most distal and medial position on the leg, with the columns of the grid in the direction of the muscle fibers, as shown in Figure 3. The grid was fixed on the skin by adhesive tape, and the reference electrode was placed on the ankle of the subject. The EMG signal was acquired at a frequency of 2048 Hz, gain of 2000, band-pass filter 10–500 Hz, and analog to digital (A/D) converted with a resolution of 12 bits. All the signals were acquired via OTBiolab+ software (OT Bioelettronica) [59]. An example of recorded EMG signals can be seen in Figure 4.

### 2.6. Data Processing

Recorded sEMG signals were processed using DEMUSE software (Maribor, Slovenia), [60] where they were off-line bandpass filtered (10–500 Hz), then decomposed with the convolution kernel compensation (CKC) technique [34,61,62,63,64]. For each contraction, 60 of the 63 acquired monopolar signals were used for the decomposition. The signals were treated separately for each contraction of each condition, each recording having a small buffer time before and after the actual contraction (the total duration of each recording was between 12 s to 15 s, and had no influence on the decomposition process). This decomposition technique has been validated extensively with both simulated and experimental signals, and the detailed analysis procedure has been described in previous studies [65,66]. Once decomposed, the firing pattern of different MUs were identified, manually inspected and edited by an experienced operator using the DEMUSE tool interface [60]. Movement artifacts and abnormally high (>30 Hz) or low (<5 Hz) instantaneous firing rate (IFR) values were discarded. Duplicates of detected MUs were also discarded. The signals that presented a pulse to noise ratio (PNR) of 30 dB or above were kept [67]. Finally, MUs with an extremely low number of firing (<15) were discarded. MUs were not tracked across the contractions, therefore, labels of MUs that were detected during the “ramp” or “ramp and maintain” contractions did not correspond to those obtained during another contraction for individual subjects. For example, MU 1 detected for one contraction was not the same as MU 1 detected for another contraction.

From those firing patterns, the following variables were computed:

- Conduction velocity (CV): The speed which an action potential travels along the membrane of a skeletal muscle fiber [68]. The estimation of the conduction velocity was calculated using the techniques described by Farina et al. [69,70], which use a maximum likelihood multiple-channel method, using the Newton method for efficient optimization. Coefficient of correlation (CC) was used to measure the goodness of match of MUAPs on neighboring HDsEMG channels and, thus, the accuracy of CV estimation. Only the CV values with corresponding CC ≥0.7 were used for the statistical analysis.
- Peak firing rate (PFR): The PFR of a MU was defined as the maximum instantaneous firing rate value for the MU. The instantaneous firing rate (IFR) of each MU was calculated:
  (1) $$IFR_{n}=\frac{1}{\left(t_{n+1}-t_{n}\right)},$$
  with $IFR_{n}$ in pps, and $t_{n+1}$ and $t_{n}$ in seconds.

**Figure 5:**
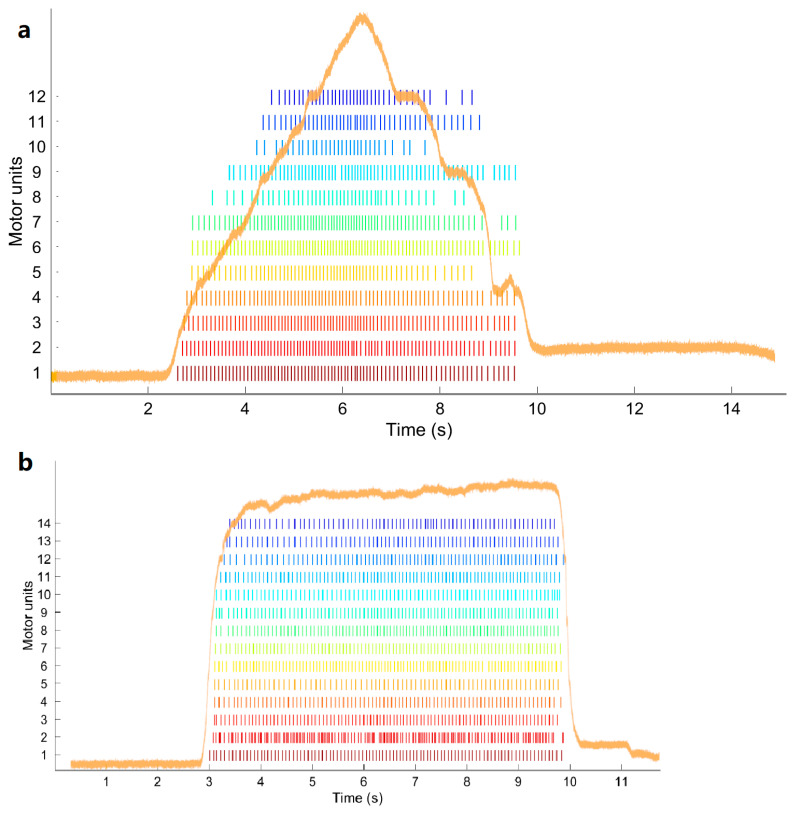
Motor unit (MU) firing time of the detected MUs for subject 2. (**a**) During the “ramp” condition at 10% MVC after control intervention. (**b**) During the “ramp and maintain” condition at 5% MVC after control intervention. A force pattern is depicted in orange.

An example of the MU firing rate is shown in Figure 5.

**Figure 6:**
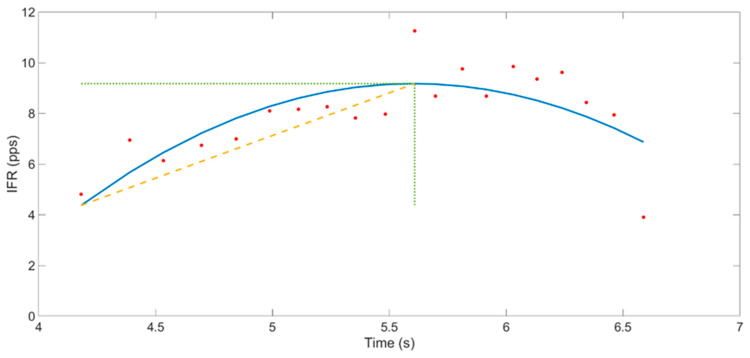
Representation of the instantaneous motor unit(MU) firing rate gradient (IFRG). The blue line depicts the 2nd order polynomial fitted to the IFR, pictured in red dots. From this polynomial, the peak IFR was retrieved (dotted green line) and then used to compute the linear expression from the first detected IFR value to the peak IFR (yellow line).

- Instantaneous MU firing rate gradient (IFRG): The IFRG is the slope of linear approximation of MU firing rate increase from recruitment to its peak IFR value (Figure 6). The following technique was used to compute this value: a polynomial of the second order was fitted on the instantaneous firing rate curve of the MU, using the polyfit() function from MATLAB 2018a (MathWorks, Natick, MA, United States). Then, a linear function was fitted on the first value and the peak value of this polynomial. The slope of the linear expression defined the IFRG. In each participant, IFRG values were averaged across contraction repetitions and across condition and intensity before being statistically analyzed.

### 2.7. Statistical Analysis

The statistical analysis was performed in R (R Foundation for Statistical Computing, Vienna, Austria) version 3.5.1 using *lme4* package version 1.1–21 [71,72] and *emmeans* package version 1.3.4. [73] Tukey’s HSD method was used to perform pairwise contrasts. Linear mixed regression or generalized linear mixed regression models were setup to investigate the influence of the chiropractic intervention on the following variables compared to the control: (i) average peak firing rate of the MUs (PFR), (ii) average instantaneous firing rate gradient of the MUs (IFRG), and (iii) average conduction velocity (CV)of the muscle fibers associated with the MUs. For each dependent variable, a separate mixed model was setup to estimate its mean and standard error across the 2 intervention sessions (Intervention: chiropractic, control) under 2 intensity levels (Intensity: 5%, 10%) in the 2 conditions (Condition: “ramp”, “ramp and maintain”). Each model was adjusted for the baseline (pre-intervention) values of the outcome variables and included a saturated fixed effects structure consisting of the independent variables and all their possible interactions. To cater for correlated repeated measures data from the same participants, the models also included random intercept effects for participants. PFR, IFRG, and CV were coded as continuous variables. Intensity, Intervention, and Condition were entered as categorical variables. A *p*-value of less than 0.05 was considered significant for all statistical tests.

## 3. Results

Fourteen participants (10 males and 4 females; age: 28.6 ± 2.8 years) participated in this study. Their baselines characteristics can be found in Table 1. Two participants (one male and one female) completed only a control session as they opted out of the study following their first assessment due to scheduling issues. Their results have, however, been included in the data processing and analysis.

Across all the subjects, a total of 2240 (Pre) and 2329 (Post) motor units were decomposed from the Control intervention session and total of 1954 (Pre-) and 1962 (Post-) motor units (MUs) were decomposed during from spinal manipulation session. In control sessions, there were 14 participants; thus, the average was 2240/14 = 160, whereas, in the experiment session, the average was 1954/12 = 162.83. Thus, the average number of MU was higher in the intervention session. The number of MU discarded because they were showcasing less than 15 firing represent 4.7% of the total MU detected (399 MU discarded for a total of 8485).

Table 2, Table 3 and Table 4 show pair-wise contrasts across sessions for the two conditions: control and intervention for instantaneous MU firing rate gradient (IFRG) of MUs, peak pulse/min of Mus, and conduction velocity (CV).

### 3.1. Conduction Velocity

**Figure 7:**
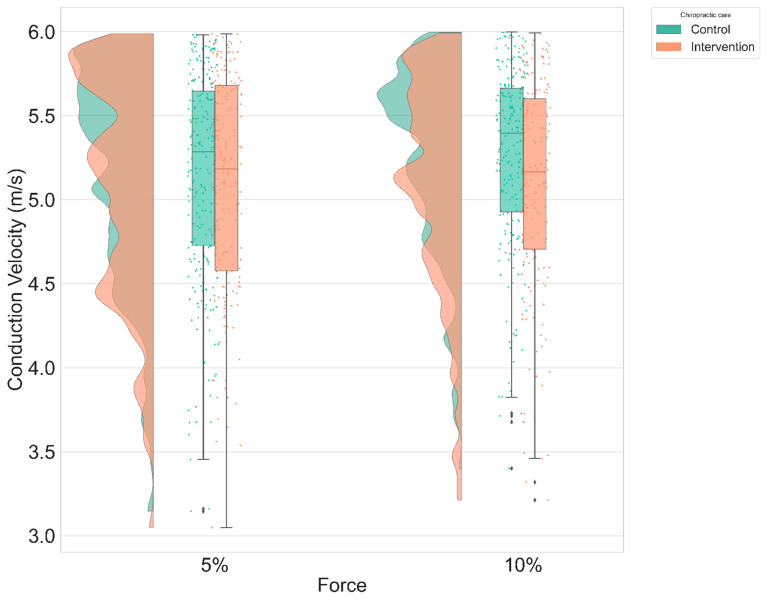
Distribution plot of the CV after intervention (control vs. spinal manipulation) for “ramp & maintain” condition adjusted to baseline.

The pair-wise contrasts across sessions for the control and chiropractic intervention indicated a statistically significant difference in the conduction velocity between control and spinal manipulation for “ramp and maintained” at 5% MVC only (0.3 ± 0.11 m.s^−1^; *p* = 0.01). The changes were not significant for the other conditions. Figure 7 shows the estimated values for the “ramp and maintain” condition between the control and spinal manipulation sessions.

### 3.2. Instantaneous MU Firing Rate Gradient & Peak Firing Rate

**Figure 8:**
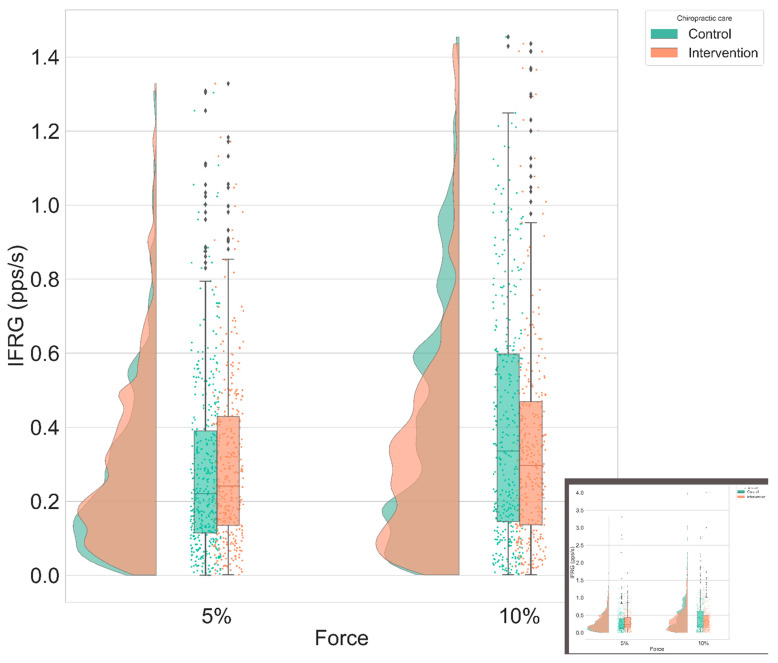
Magnified group distribution plot of the IFRG after intervention (control vs. spinal manipulation) for “ramp & maintain” condition adjusted to baseline. (Unmagnified version is in Appendix A)

**Figure 9:**
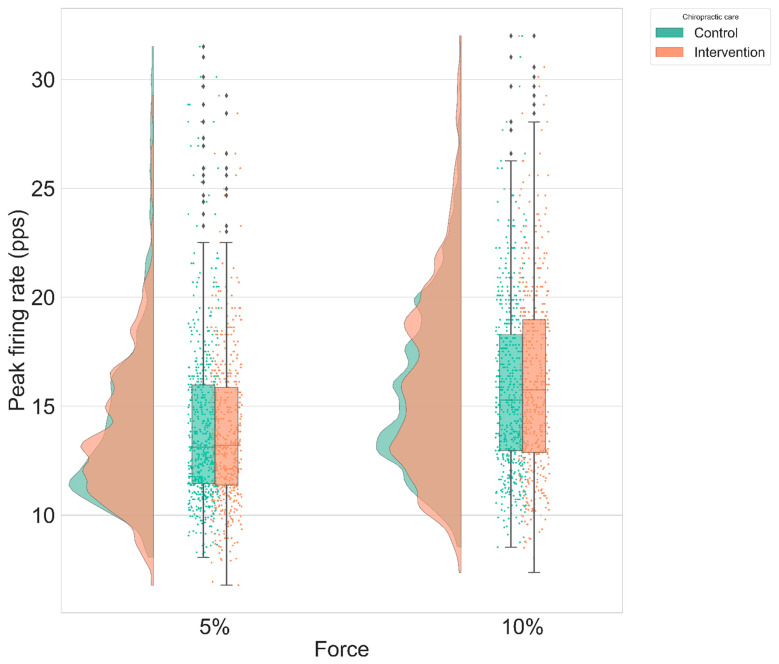
Group distribution plot of the PFR after intervention (control vs. spinal manipulation) for “ramp & maintain” condition adjusted to baseline.

No significant changes were found for the instantaneous MU firing rate gradient, and peak firing rate variables. Table 3 and Table 4 summarize the results, respectively, for instantaneous MU firing rate gradient and peak firing rate. The following figures display the distribution plots of the results for instantaneous MU firing rate gradient for the condition “ramp & maintain” in Figure 8 and for peak firing rate for the condition “ramp & maintain” in Figure 9.

## 4. Discussion

The results revealed a main effect of spinal manipulation on the conduction velocity of the MUs. This supports previous studies that have suggested that spinal manipulation has an impact on sensory and motor neural processing that impacts motor control [3,4,5,6,7,74]. This study aimed to evaluate the influence of spinal manipulation on MU activity and therefore, neuromuscular control. To achieve this aim, we used HD sEMG with advanced signal processing techniques to obtain information regarding MU behavior. Based on previous research involving spinal manipulation, we expected a decrease in conduction velocity and/or an increase in instantaneous MU firing rate gradient and peak firing rate following the chiropractic intervention. Each of these changes could explain neuromuscular adaptions previously documented after spinal manipulation [1,2,3,7,8,9,10,11].

The results showed significant changes only in the “ramp & maintain” condition at 5% of the MVC, where the conduction velocity decreased in the spinal manipulation session as compared to the control session. It is known that conduction velocity of an MU is correlated to its twitch torque production capacity [35]; therefore, the decrease of conduction velocity after spinal manipulation would reflect the recruitment of MU producing, on average, lower twitch torque. This decrease in conduction velocity after spinal manipulation suggests that spinal manipulation increases the activation of lower threshold MUs which are better suited for the production of low force and precise action during low contraction level of the TA muscle.

A recent study [74] explored the effects of a single spinal manipulation session on maximum voluntary contractions (MVC) of the ankle dorsiflexors, HD EMG, intramuscular EMG, and near-infrared spectroscopy (NIRS) from the TA muscle in 25 participants with low level recurring spinal dysfunction. They found a significant increase in conduction velocity in both the isometric steady-state contractions at 10% of MVC and during the isometric ramp contractions at 10% of MVC compared to the control intervention [74]. The increased MVC in TA strength and increase in conduction velocity, without changes in motor unit discharge rate, suggests that the MVC strength changes observed following spinal manipulation in this study were mostly due to increased recruitment of larger, higher threshold motor units. It is interesting to note that this recent study found an increase in conduction velocity at 10% MVC following the spinal manipulation session, whereas, in the current study, using the HD sEMG decomposition methodology, we found a decrease in the conduction velocity at 5% MVC [74]. Both studies recorded EMG during similar “ramp” and “ramp & maintain” conditions at both 5% and 10% MVC [74]. This apparent contradiction in finding may be due to the difference in the way the conduction velocity across the TA was calculated. In a recent study [74], the conduction velocity was calculated using algorithms of cross-correlation and a maximum likelihood multi-channel approach adapted from Farina et al. [75]. This method involved iteratively cross-correlating signals in a multi-channel matrix to find the electrodes whose signals had the least mean square error while calculating the similarity between them. In the current study, we used the HDsEMG decomposition methodology and calculated the conduction velocity using the techniques described by Farina et al. in 2000 and 2001, respectively [69,70], which use a maximum likelihood multiple-channel method, using the Newton method for efficient optimization. Coefficient of correlation (CC) was used to measure the goodness of match of MUAPs on neighboring HDsEMG channels and, thus, the accuracy of CV estimation. Only the CV values with corresponding CC ≥0.7 were used for the statistical analysis. These methodological differences may explain the differences in conduction velocity changes following spinal manipulation. Alternatively, it could be individual variation in responses to the spinal manipulations themselves that could explain these differences. However, the most likely explanation is that the two different methods for calculating CV are using different strategies. One method is measuring CV from decomposed single Mus, and the other is measuring the CV of all MUs under the HDsEMG electrode grid. Regardless, it appears that spinal manipulation alters the central corticomotor control of the TA muscle, possibly by increased recruitment of larger, higher threshold motor units to enable greater levels of MVC, and/or by increases the activation of lower threshold MUs which are better suited for the production of low force and precise action as required by the 5% “ramp” and “ramp & maintain” conditions required of the subjects in this study.

Further studies should explore these finding in higher percentage of MVC contractions, and carefully consider any significant changes of the IFRG induced by spinal manipulation. Indeed, as it is developed in the theories on MU recruitment, be it the dominant theory of Kernell [76,77] or its main alternative proposed by De Luca and Contessa [78], the IFRG is correlated to the MUs excitation threshold; thus, the IFRG could yield valuable information. No significant results concerning the IFRG could be found in our study, but regarding the data acquired, it seems that the choice of the type of force production pattern could impact the IFRG as we can see opposite changes between the condition “ramp” and “ramp & maintain” were the IFRG seems to, respectively, increase or decrease. Therefore, the choice of the force production patterns should be carefully considered in future studies. It is interesting to note this trend for a task-specific change in IFRG between “ramp” and “ramp & maintain” conditions following spinal manipulation. A previous study has shown that spinal manipulation can have complete opposite neuromuscular changes to two different functional muscles [14]. This study found that, after spinal manipulation, there was a decrease in short interval intracortical inhibition, an increase in intracortical facilitation, and a shortening of the cortical silent period in the flexor muscle, whereas the extensor muscle showed opposite changes, with a decrease in intracortical facilitation and a lengthening of the cortical silent period [14]. It may be that spinal manipulation can also alter corticospinal excitability to the same muscle in a different direction depending on the task required (i.e., “ramp” and “ramp & maintain” in the current study). This should be explored further in future studies.

Multiple previous studies have documented strength increases following a spinal manipulation intervention. One study demonstrated a 16% increase in plantar flexor muscle strength after a single session of spinal manipulation in subclinical pain participants [19]. Another study found an 8% increase in plantar flexor muscle strength following a single session of spinal manipulation in elite taekwondo athletes [3]. A third study was conducted in a group that had lost their ability to cortically activate their muscles, i.e., chronic stroke patients with ongoing plantar flexor muscle weakness which found that spinal manipulation significantly increase their plantarflexion muscle strength by on average 64.2% [7]. This is rather remarkable and suggests spinal manipulation may be of great value for both athletes and those who have lost their cortical ability to activate their muscles. This should be further explored in future studies. The current study finding that showed changes in in the conduction velocity MU level suggests that spinal manipulation may induce task dependent changes and also help to elucidate the exact mechanisms of how spinal manipulation can impact human motor control.

## 5. Limitation

There are multiple limitations to consider for this study. The force production patterns were chosen in order to “isolate” lower threshold MUs that were believed to be impacted by spinal manipulation [19,55,56,57]. However, it is important to keep in mind that high-threshold motor units are also very likely to change following spinal manipulation [10] which the current study design in unable to elucidate. The current study design only allowed us to extract the lower threshold MU activity patterns that were executed at low force levels. In addition, note that the non-significant change in instantaneous MU firing rate gradient between “ramp” and “ramp & maintain” condition are only trends as no significant differences in the alterations from spinal manipulation were seen between the two patterns. This may be because both conditions were the same type of contraction, isometric, and executed at low force. The lack of any significant difference in the results between the different conditions may, therefore, be that they were too similar. Each individual was also adjusted according to his or her spinal dysfunction pattern, and this may have influenced the homogeneity of the results and, thus, the outcome. Each subject was adjusted at different spinal levels so the degree to which their particular spinal dysfunction pattern, and thus the spinal manipulations they received for it, was impacting the motor control of the tibialis anterior may have varied. In addition, we have to take into account that the spinal manipulation provided in this study was only a single session. Some neurophysiological changes observed after spinal manipulation have been shown to require multiples sessions over time [6].

Gender may have influenced the outcome of this study, although gender has been shown to influence motor control more in an elderly population [79] than the participants of the current study. This could be followed up in future studies, as the current sample size does not allow for such factorial analysis.

## 6. Conclusions

This study shows that spinal manipulation provides at least short-term benefits in low intensity movement, via the improvement of the recruitment of low threshold motor unit and low twitch torque that are better suited for low force precise tasks. Further research is required to investigate the longer term and potential functional effects of spinal manipulation and chiropractic care in a variety of patients who would benefit from improved muscle function. Future studies should also use higher force levels to investigate the effects of spinal manipulation on motor unit behavior as EMG decomposition techniques have improved and now also allow for this type of analysis.

## Figures and Tables

**Table 1:** Baseline measurements before Intervention (mean ± SE)**.**

	Control Group (*N* = 14)	Intervention Group (*N* = 12)
Conduction velocity (m.s^−1^)	5.11 ± 0.62	5.12 ± 0.61
Peak firing rate (pps)	15.79 ± 4.04	15.45 ± 3.9
Instantaneous MU firing rate gradient (pps.s^−1^)	1.33 ± 1.48	1.37 ± 1.51

**Table 2:** Estimated Group differences in the conduction velocity (CV) across groups.

Condition, Intensity	Control—Intervention Difference (Mean ± SE)	*df*	T Ratio	*p* Value
Ramp and maintain, 5%	0.3 ± 0.11 m.s^−1^	53.7	2.64	0.01
Ramp and maintain, 10%	0.2 ± 0.12 m.s^−1^	53.8	1.65	0.11
Ramp, 5%	0.07 ± 0.11 m.s^−1^	53.8	0.66	0.51
Ramp, 10%	0.03 ± 0.12 m.s^−1^	54.4	0.28	0.78

**Table 3:** Estimated group differences in the average instantaneous MU firing rate gradient (IFRG) of MUs across groups.

Condition, Intensity	Ratio Control/Intervention Difference (Mean ± SE)	*z*-Value	*p*-Value
Ramp and maintain, 5%	0.99 ± 0.15	−0.05	0.96
Ramp and maintain, 10%	0.95 ± 0.15	−0.32	0.75
Ramp, 5%	1.27 ± 0.2	1.52	0.13
Ramp, 10%	1.174 ± 0.19	0.98	0.33

**Table 4:** Estimated group differences in the average peak firing rate (PFR) of MUs across groups.

**Condition, Intensity**	**Control—Intervention** **Difference (Mean ± SE)**	***z*** **-Value**	***p*** **-Value**
Ramp and maintain, 5%	0.286 ± 0.4 pps	0.715	0.47
Ramp and maintain, 10%	−0.24 ± 0.47 pps	−0.508	0.61
Ramp, 5%	0.14 ± 0.42 pps	0.33	0.74
Ramp, 10%	0.1 ± 0.52 pps	0.2	0.84

**Figure A1:**
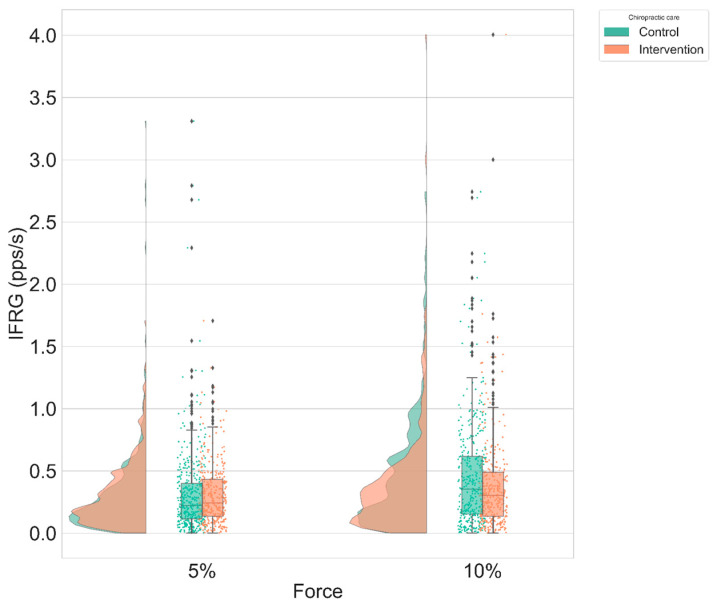
Unmagnified distribution plot of the IFRG after intervention (control vs. spinal manipulation) for “ramp & Maintain” condition adjusted to baseline (Unmagnified Figure 8).

## Data Availability

The data are not publicly available because of ethics committee restriction.
